# Odor Identification Test in Idiopathic REM-Behavior Disorder and Parkinson's Disease in China

**DOI:** 10.1371/journal.pone.0160199

**Published:** 2016-08-02

**Authors:** Si-Fei Huang, Kui Chen, Jian-Jun Wu, Feng-Tao Liu, Jue Zhao, Wei Lin, Si-Si Guo, Yi-Xuan Wang, Ying Wang, Su-Shan Luo, Yi-Min Sun, Zheng-Tong Ding, Huan Yu, Jian Wang

**Affiliations:** 1 Department of Neurology, Huashan Hospital, Fudan University, Shanghai, China; 2 Department of Neurology, Guangdong Neuroscience Institute, Guangdong General Hospital, Guangdong Academy of Medical Sciences, Guangzhou, Guangdong Province, China; 3 Sleep and Wake Disorders Center of Fudan University, Shanghai, China; Oasi Institute for Research and Prevention of Mental Retardation, ITALY

## Abstract

**Background:**

Olfactory dysfunction is common in Parkinson's disease (PD) and idiopathic rapid eye movement sleep behavior disorder (iRBD), which is a risk factor in the development of PD. However, a few studies have conflicting results when comparing dysosmia in the patients with iRBD and PD. There is no study investigating the olfactory function in Chinese patients with iRBD. Additionally, the Sniffin’ Sticks screening 12 test (SS-12) contains several odors that are not familiar to people in different cultures.

**Methods:**

Odor identification was evaluated in iRBD patients (n = 54), PD patients (n = 54) and healthy controls (n = 54). With the identification data, a brief odor identification test was established and then validated in other subjects.

**Results:**

Odor identification scores in iRBD patients were significantly higher than those in PD patients (*P*<0.001) but lower than those in controls (*P*<0.001). At the cut-off value of 7.5, the Sniffin’ Sticks clearly differentiated iRBD and PD patients from the controls, and the brief test could increase the specificity in diagnosing PD. Neither the Sniffin’ Sticks nor the brief test could clearly differentiate PD and iRBD patients from each other.

**Conclusions:**

Olfaction is more impaired in PD patients than in iRBD patients, possibly due to the heterogeneity of iRBD patients. The Sniffin’ Sticks could be a useful tool for differentiating iRBD patients from the healthy population, and it could be useful for screening people at high-risk of PD in China, especially when combined with polysomnography. To reduce the expense and time required for the Sniffin’ Sticks test, this study shows that a brief test is feasible.

## Introduction

Parkinson disease (PD), clinically characterized by tremor, bradykinesia, rigidity and postural instability, is a multisystem disease with variegated non-motor (NM) deficits, including impaired olfaction, sleep disorders and neuropsychiatric disorders [1]. PD affects more than 1.7 million Chinese people over 55 years of age [2]. However, when a clinical diagnosis of PD is made, there has already been an extensive loss of dopaminergic neurons in the substantia nigra (SN) [3]; thus, it is important to diagnose PD early to retard the progression of disease. In more than 90% of PD patients, olfactory loss is found preceding the motor symptoms [4], and the high prevalence of olfactory loss and its early occurrence suggests that an olfactory test can be a supplementary diagnostic tool for PD.

Rapid eye movements sleep behavior disorder (RBD) is a parasomnia characterized by the intermittent loss of REM sleep electromyographic (EMG) atonia and dream-enacting behavior [5, 6]. It is one of the non-motor symptoms (NMS) of PD that may precede the onset of motor symptoms of PD patients [7]. Studies have reported that RBD occurs in approximately 30–60% of patients with PD [8], and 50–90% of idiopathic RBD (iRBD) patients eventually developed PD or dementia with Lewy Bodies (DLB), or other synucleinopathies [9–11], indicating that a considerable proportion of iRBD patients are in the prodromal phase of PD. These findings justify studying the features of prodromal PD in patients with iRBD.

As prodromal symptoms of PD, olfactory disorder and iRBD are supposed to be relevant to each other. Several studies have reported substantial olfactory impairment in iRBD patients [12–20], with a wider range of reported olfactory impairment compared to PD patients. One study suggested that olfaction was more impaired in PD patients than that in iRBD patients [16], while other studies found no differences in olfaction between PD and iRBD patients [15, 17, 20]. In a recent study, the Sniffin' Sticks odor identification test successfully differentiated PD patients from healthy controls [21], but the conflicting results across studies suggests that the degree of olfactory dysfunction in iRBD is still unclear, especially compared with PD. These mixed results have caused more uncertainty about the using the odor identification test as an identification tool for prodromal PD, and it remains unknown whether the odor identification test can be applied in this aspect in China.

What’s more, the primary design of the Sniffin' Sticks screen 16 test is mainly based on the odor identification rate in American people [22]. Although there is an officially adapted version for Asian populations, the cultures are so different in various countries that it may not fit every culture; that is why there are different versions of the Sniffin’ Sticks test in different countries, such as Estonia and Japan [23, 24], to improve its diagnostic value. For the Chinese, several odors in the Sniffin’ Sticks are not appropriate as well, such as cinnamon and cloves. Additionally, the existing Sniffin’ Sticks test has some other drawbacks in terms of the detection costs and the average time cost in practice. In light of these factors, we hope to develop more a simplified version of the Sniffin’ Sticks test to generalize the use of this olfactory test to diagnose prodromal PD in China.

In this research, we assessed the differences in olfactory identification among iRBD patients, PD patients and healthy controls using the Sniffin’ Sticks screening 12 test (SS-12) [25], and we evaluated the application of the odor identification test as a supportive tool for screening people at high-risk of PD in the Chinese population. Additionally, we piloted a brief odor identification test to verify its application value. Because olfactory function may be influenced by age, sex and cultural differences, we employed a matched case-control study design to avoid these confounding factors.

## Materials and Methods

### Subjects

Fifty-four iRBD patients were consecutively recruited from the somnology center at the Department of Neurology, Huashan Hospital affiliated with Fudan University, Shanghai, China, between November, 2013 and November, 2014 to take the Sniffin’ Sticks screening 12 test. Thirty-five iRBD patients were recruited from the same site between January, 2015 and July, 2015 to take the brief odor identification test. All of the patients had a history of dream-enacting behaviors [6] and video polysomnographic confirmation of increased electromyographic activity during rapid eye movement (REM) sleep associated with abnormal behaviors [26].

Patients with idiopathic PD who met the United Kingdom PD Society Brain Bank criteria [27] were recruited from the movement disorders clinic. Through a RBD Single-Question Screen [28] and a history about sleep, patients with sleep behavior disorder were excluded. Fifty-four age (±5 years), sex and education-paired subjects without other neurological diseases were selected from those patients between November, 2013 and November, 2014. Thirty-five age (±5 years) and sex-paired subjects were selected in the same way between January, 2015 and July, 2015. During these two periods, fifty-four age (±5 years), sex and education-paired and thirty-five age (±5 years) and sex-paired healthy controls with no symptoms or history of RBD were recruited from the general population in Shanghai separately.

Subjects were excluded if they had cognitive impairment, if their Mini-Mental State Examination (MMSE) score was less than 24, and if they had other factors that may influence olfactory function, including head trauma, nasal surgery, acute or chronic upper respiratory tract infection. The study was approved by the ethics committee of Huashan Hospital and all participants signed informed consent.

### Clinical assessment

All participants received a questionnaire containing age, gender, etc., to collect demographic information, and they underwent a systematic medical history of symptoms, signs, rest hours, organic disease history, etc. They also had a complete neurological examination including a physical examination of the nervous system and a radiological examination of the head by CT or MRI to exclude neurodegenerative diseases and the lesions in the brainstem. IRBD patients were monitored by polysomnography (PSG) all night in the somnology center, and PD patients were assessed for disease severity using the Hoehn and Yahr modified staging scale [29]. All participants were evaluated for cognitive function by MMSE [30].

### Olfactory assessment

Odor identification was assessed for both nostrils using the Sniffin’ Sticks screening 12 test [25] in 54 iRBD patients, 54 PD patients and 54 healthy controls, and using a brief odor identification test in 35 iRBD patients, 35 PD patients and 35 healthy controls. The standard methodology for these tests was followed, with assurance that the felt-tip pens were innocuous, nonpoisonous and used with odorless and often replaceable gloves. This test required the participants to refrain from eating or drinking 15 mins before the test. Briefly, in this progress, the felt-tip pens presenting odors were consecutively placed approximately 2 cm in front of both nostrils, with a 20-s interval between odor presentations. For each odor, the participants were forced to choose the correct odor from a list of 4 descriptors. Each correct answer received a score of 1, and the identification score ranges from 0 to 12.

### Statistical analysis

Median and inter-quartile range (IQR) were used to describe the distribution of continuous variables. Since the patients were paired among three groups, they were compared using the Friedman test for continuous variables and Cochran’s Q test for categorical data. Post-hoc analysis was conducted with Bonferroni correction applied. Receiver operating characteristic (ROC) analysis was used to calculate the respective optimal cut-off values of the identification score. The significance level was set at P<0.05. Statistical analyses were performed with the IBM SPSS Statistics Standard Software (version 22.0).

## Results

### Description of the study groups

There was no significant difference in age, gender distribution and education level among the three groups (Table 1). The median duration of disease for iRBD and PD were 34.5 months and 32.5 months, respectively. In PD patients, 44 (81.5%) were at H & Y stage 1–2, with 10 (18.5%) at stage 3. More clinical information could be found in S1 and S2 Tables.

**Table 1:** Patient Demographics and Odor Identification Performance.

	HC (N = 54)	iRBD (N = 54)	PD(N = 54)	YI	*P* value	Post-hoc significance
Age^a^ (years)	65(61,70)	65(60,70)	65(60,70)		0.189	N/A
Gender^b^ (Male/Female)	43/11	43/11	43/11		1.000	N/A
Education^b^ (> = 9yrs/<9yrs)	51/3	48/6	50/4		0.097	N/A
Duration of disease (Months)	/	34.50(14.50,80.75)	32.50(12.75,50.50)			N/A
H & Y stage (1-2/3)	/	/	44/10			
Identification scores^a^	9(8,10)	6(5.75,7)	4(3,6.25)		**<0.001**	HC>iRBD***,HC>PD***,iRBD>PD***
Identification rates						
Orange^b^	87.0%	68.5%	40.7%	0.463	**<0.001**	HC>PD***, iRBD>PD**
Leather^b^	72.2%	42.6%	38.9%	0.333	**0.002**	HC>iRBD*, HC>PD**
Cinnamon^b^	46.3%	29.6%	31.5%	0.148	0.154	N/A
mint^b^	92.6%	59.3%	46.3%	0.463	**<0.001**	HC>iRBD***, HC>PD***
Banana^b^	75.9%	42.6%	37.0%	0.389	**<0.001**	HC>iRBD**, HC>PD***
Lemon^b^	63.0%	35.2%	35.2%	0.278	**0.003**	HC>iRBD**, HC>PD*
Liquorice^b^	63.0%	50.0%	25.9%	0.370	**<0.001**	HC>PD***, iRBD>PD*
Coffee^b^	94.4%	75.9%	37.0%	0.574	**<0.001**	HC>iRBD*, HC>PD***,iRBD>PD***
Clove^b^	59.3%	59.3%	37.0%	0.222	**0.03**	
Pineapple^b^	75.9%	48.2%	33.3%	0.426	**<0.001**	HC>iRBD*, HC>PD***
Rose^b^	77.8%	33.3%	46.3%	0.315	**<0.001**	HC>iRBD***, HC>PD**
Fish^b^	90.7%	64.8%	48.2%	0.426	**<0.001**	HC>iRBD*, HC>PD***

Data for continuous variables presented as medium (inter-quartile range).

^a^ *P* value calculated using Friedman test.

^b^ *P* value calculated using Cochran’s Q test followed by Bonfferoni’s test.

Values in bold refer to statistically significant difference (*P*<0.05)

*P<0.05

**P<0.01

***P<0.001

Abbreviations: HC = Healthy Control; iRBD = idiopathic REM sleep behavior disorder; PD = Parkinson’s disease; H & Y stage = Hoehn and Yahr stage; YI = Youden’s Index; N/A = not applicable.

### Odor identification by Sniffin’ Sticks screening 12 test in controls, iRBD patients and PD patients

**Fig 1:**
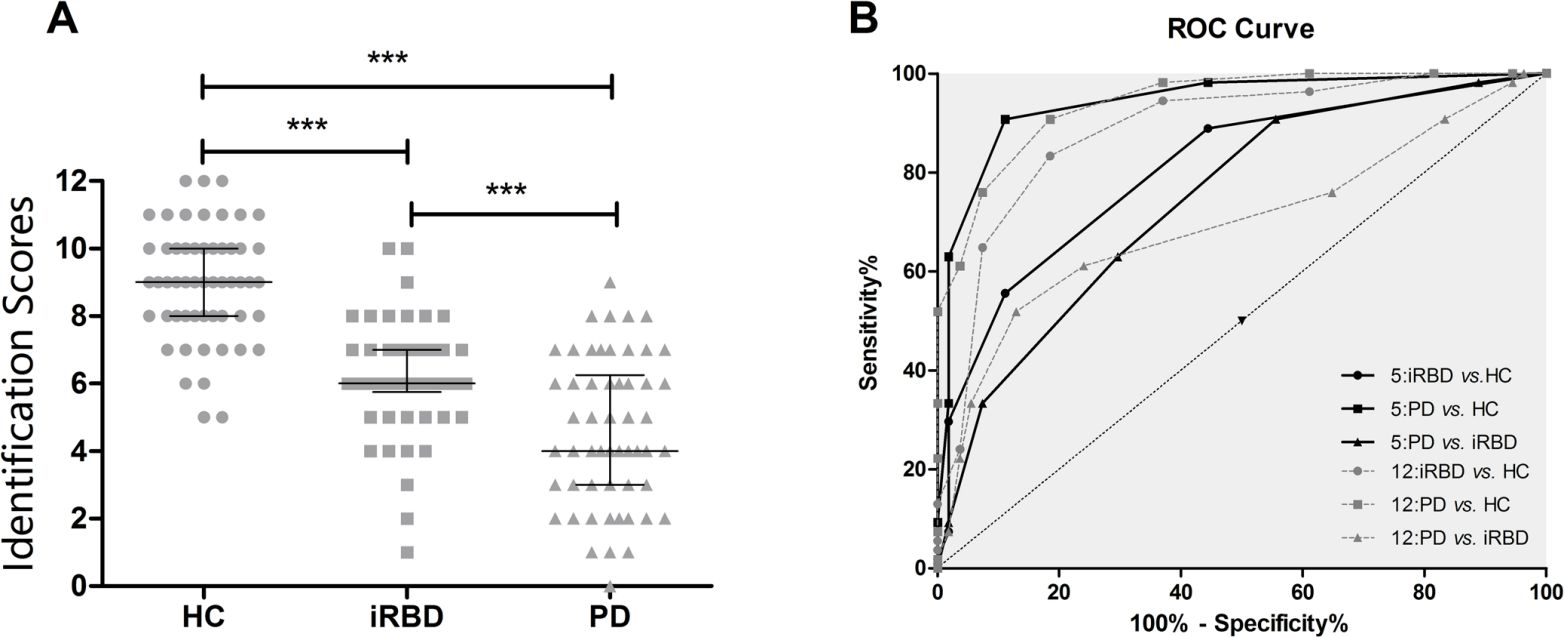
Differentiation among HC, iRBD and PD patients by the odor identification tests. (A) Scatterplots of individual scores with the respective group median and 25th and 75th percentiles for the Sniffin’ Sticks. (B) Receiver Operating Characteristic (ROC) curves showing the relationship between sensitivity and specificity of the Sniffin’ Sticks (dotted lines) and the brief test (solid lines). ***P<0.001

Odor identification scores varied significantly across the groups (Table 1). Median (IQR) identification scores were 9 (8, 10) in controls, 6 (5.75, 7) in iRBD patients and 4 (3, 6.25) in PD patients. Total scores were significantly lower in the iRBD (*P*<0.001) and PD (*P*<0.001) groups than in the control group, and PD patients had lower identification scores than the iRBD group (*P*<0.001) (Fig 1A).

For odor identification, a cut-off value of 7.5 in SS-12 best differentiated PD patients from controls as shown in the ROC curve (Table 2, Fig 1B), which reveals 90.7% sensitivity and 81.5% specificity (AUC: 0.941, 95% CI: 0.902–0.981, *P*<0.001). With the same cut-off value, differentiating between patients with iRBD and healthy controls, the sensitivity is 83.3% and specificity is 81.5% (AUC: 0.885, 95% CI: 0.820–0.950, *P*<0.001). However, the optimal cut-off value calculated by ROC analysis for separating iRBD and PD is 4.5 rather than 7.5, with a sensitivity of 51.9% and a specificity of 87.0% (AUC: 0.699, 95% CI: 0.596–0.798, *P*<0.001).

**Table 2:** Discriminant Analysis for the Odor Identification Tests.

	ROC AUC (95% CI)	*P* value	Cut-off Value	Sensitivity	Specificity
The Sniffin' Sticks screening 12 test					
iRBD vs. HC	0.885 (0.820–0.950)	**< 0.001**	7.5	83.3%	81.5%
PD vs. HC	0.941 (0.902–0.981)	**< 0.001**	7.5	90.7%	81.5%
PD vs. iRBD	0.699 (0.596–0.798)	**< 0.001**	4.5	51.9%	87.0%
The brief odor identification test					
iRBD vs. HC	0.806 (0.723–0.882)	**< 0.001**	3.5	55.6%	88.9%
PD vs. HC	0.940 (0.894–0.986)	**< 0.001**	3.5	90.7%	88.9%
PD vs. iRBD	0.744 (0.652–0.836)	**< 0.001**	3.5	90.7%	44.4%
The validation of the brief odor identification test					
iRBD vs. HC	0.822(0.726–0.918)	**< 0.001**	3.5	64.1%	89.7%
PD vs. HC	0.956 (0.914–0.998)	**< 0.001**	3.5	92.3%	89.7%
PD vs. iRBD	0.781 (0.680–0.882)	**< 0.001**	3.5	92.3%	35.9%

Values in bold refer to statistically significant difference (*P*<0.05).

Abbreviations: HC = Healthy Control; iRBD = idiopathic REM sleep behavior disorder; PD = Parkinson’s disease; ROC AUC = receiver operating characteristic area under curve.

Odorants in Sniffin’ Sticks differed with each other not only in identification rates in healthy controls but also in the accuracy of discriminating iRBD or PD patients from controls. The odor of coffee, mint and fish were correctly identified by more than 90% of controls, while the odor of cinnamon and clove were frequently not identified by controls (<60%) (Table 1). Except for cinnamon, all items showed statistically significant differences among the three groups in identification rates. With the exception of cinnamon and clove, the identification rates of all single items were significantly lower in PD patients than in controls. The odor of coffee, orange, mint, pineapple and fish showed the largest differences between PD patients and controls.

### Odor identification by the brief odor identification test in controls, iRBD patients and PD patients

All of the odors, except cinnamon, successfully differentiated iRBD and PD patients from controls (Table 1). After Youden’s Index was calculated for differentiating PD patients from controls, coffee, orange, mint, pineapple, fish, banana and liquorice, 7 odors in total, had high indices (>0.350), which meant they had high accuracy in discriminating PD patients and controls. Among these 7 odors, coffee, orange, mint, pineapple and fish were identified correctly by more than 75% of the healthy controls and were the top-5 odors to compose the brief odor identification test. The brief test showed similar accuracy in separating PD patients from controls (AUC: 0.940, 95% CI: 0.894–0.986, *P*<0.001), but had higher accuracy in differentiating PD from iRBD patients (AUC: 0.806, 95% CI: 0.723–0.882, *P*<0.001) in contrast with Sniffin’ Sticks (Table 2, Fig 1B). However, the brief test performed worse than the Sniffin’ Sticks test in separating iRBD patients from controls (AUC: 0.744, 95% CI: 0.652–0.836, *P*<0.001). In the 5-item set, a sensitivity of 55.6% and a specificity of 88.9% were achieved for separating iRBD patients from controls, with the cut-off value at 3.5; the sensitivity was higher (90.7%), and the specificity was the same when differentiating PD patients from controls with the same cut-off value. For discriminating iRBD and PD patients, the sensitivity was 90.7%, and the specificity was 44.4%.

### Validation of the brief odor identification test

**Fig 2:**
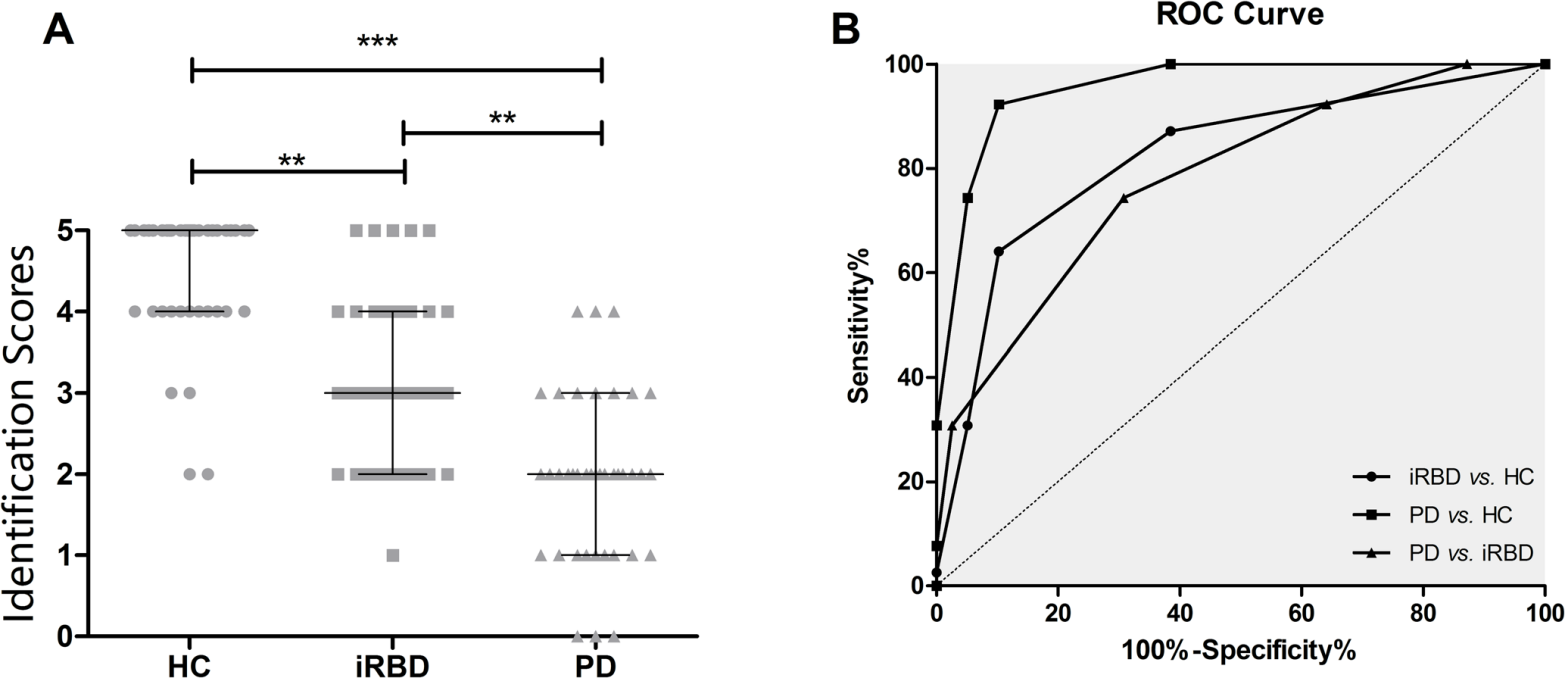
Differentiation among HC, iRBD and PD patients by the brief test in validation. (A) Scatterplots of individual scores with the respective group median and 25th and 75th percentiles for the brief test in validation. (B) Receiver Operating Characteristic (ROC) curves showing the relationship between the sensitivity and specificity of the brief test in validation. ***P<0.001, **P<0.01

To confirm the value of the brief test, further validation test was undertaken. There was no significant difference in age and gender distribution among the three groups (Table 3). The median duration of disease was 50.50 and 76.50 for iRBD and PD patients, respectively. In the PD group, 21(52.5%) were at H & Y stage 1–2, and 19(47.5%) were at stage≥3. In general, the characteristics of the patients in this validation test were similar to those in the Sniffin’ Sticks test. After the brief test, the median (IQR) identification scores were 5(4, 5) in controls, 3(2, 4) in iRBD patients and 2(1, 3) in PD patients, with significant differences among the three groups (Fig 2A). The scores were significantly higher in the iRBD group than in the PD group (*P*<0.05), but were lower than in controls (*P*<0.001). The total scores in the PD group were also significantly higher than in controls. In separating patients with iRBD and healthy controls using the brief test, the sensitivity was 67.5%, and the specificity was 92.5% (AUC: 0.873, 95%CI: 0.792–0.951, *P* <0.001), with a cut-off value of 3.5 (Table 2, Fig 2B). When differentiating PD from controls, the sensitivity was higher (82.5%), but the specificity was the same. In separating PD and iRBD, the sensitivity and specificity was 82.5% and 32.5%, respectively.

**Table 3:** Patient Demographics in the Validation Test.

	HC(N = 35)	iRBD(N = 35)	PD(N = 35)	*P* value	Post-hoc significance
Age^a^ (years)	65(62,72)	66(63,71)	67(63,72)	0.269	N/A
Gender^b^ (Male/Female)	27/8	27/8	27/8	1	N/A
Duration of disease (Months)	/	49(24,92)	75(23,118)	N/A	N/A
H & Y stage (<3/> = 3)	/	/	19/16		
Identification Scores^c^	5(4,5)	3(2,4)	2(1,3)	<0.001	HC>iRBD***,HC>PD***, iRBD>PD**

Data for continuous variables presented as medium (inter-quartile range).

Values in bold refer to statistically significant difference (*P*<0.05).

^a, c^ *P* value calculated using Friedman test.

^b^ *P* value calculated using Cochran’s Q test.

**P<0.01

***P<0.001

Abbreviations: HC = Healthy Control; iRBD = idiopathic REM sleep behavior disorder; PD = Parkinson’s disease; H & Y stage = Hoehn and Yahr stage; N/A = not applicable.

## Discussion

A previous study reported the good application value of Sniffin’ Sticks screening 16 test to differentiate PD patients from controls [21]. The present study shows that the Sniffin’ Sticks screening 12 test can differentiate iRBD patients and PD patients from healthy controls successfully as well.

Thus far, we have confirmed that this was the first report to assess odor identification in Chinese iRBD patients; in this study, we not only assessed the odor identification of iRBD patients but also compared it with that in PD patients and in normal controls, finding significant differences among them. Our results verified that olfactory impairment is common in iRBD, but to a lesser degree than in PD. Our findings disagreed with some previous studies reporting no differences in odor identification between PD patients and iRBD patients [15, 17, 20]. The results from these studies are based on different methods of the Odor Stick Identification Test for the Japanese (OSIT-J) and the 12-item Cross-Cultural Smell Identification Test (CC-SIT) for olfactory identification, and these study populations also had some unmatched items, such as education levels, which can influence olfactory function significantly [31]. Our study avoided confounding factors by employing a matched case-control study design, and our findings were supported by a similar study by the University of Pennsylvania Smell Identification Test (UPSIT) comparing the two groups with age and sex adjusted olfaction scores [16].

This study revealed that significant olfactory impairment could be observed in patients with iRBD and PD more frequently compared to healthy controls. With the same cut-off value of 7.5, both iRBD and PD patients could be well discriminated from controls through the Sniffin’ Sticks screening 12 test, for which the sensitivity and specificity was 83.3% and 81.5% for iRBD, respectively, and 90.74% and 81.5% for PD, respectively, indicating that the SS-12 could be a useful tool to help diagnose iRBD and PD in China.

However, the SS-12 showed lower sensitivity in separating iRBD than PD from controls, a result that may be caused by the heterogeneity of iRBD. Further findings through a prospective study revealed that the iRBD patients with impaired olfaction were at higher risk of developing a certain type of neurodegenerative disease than those with normal olfaction [32], but there is no definite trend of its development. It could evolve into PD, multiple system atrophy (MSA) or dementia of Lewy bodies (DLB) [33], which are related to olfactory impairment at different levels [34–36] so that iRBD patients cannot be identified very accurately by olfaction tests such as Sniffin’ Sticks. On the other hand, because different paths of evolvement of iRBD are related to different levels of olfactory impairment, we may also predict the development of iRBD through the level of olfactory impairment.

Recent studies suggested that the degree of olfactory dysfunction in iRBD patients is relatively stable in at least a 4–5 year-period before some iRBD patients develop a neurodegenerative disease [11, 32]. During the process of developing clinical PD, the olfactory function of iRBD patients does not seem to worsen. It may be explained by the earlier impairment of olfaction relative to the impairment of rapid eye movement (REM) sleep in the course of disease, so the hyposmia could reach the floor effect before RBD is diagnosed. This possible disease progression is in agreement with the Braak staging for Lewy pathology in sporadic PD that olfactory impairment (stage 1) precedes the onset of sleep disorders including iRBD (stage 2) [37], which offers theoretical foundations for the low accuracy in differentiating iRBD patients from PD patients by the odor identification test.

As is mentioned above, olfactory impairment and sleep disorders emerge before motor symptoms become apparent, but ahead of that, neurodegeneration of dopaminergic neurons in the substantia nigra have taken place [38]. That is why early intervention might be more likely to be necessary and effective for PD, and offer a hypothesis that the combination of olfactory testing and reported symptoms of RBD could be useful in diagnosing prodromal PD, increasing the likelihood for early detection and intervention. Researchers found that olfactory impairment and RBD occurs in more than 90% [4] and nearly 50% [39, 40] of PD patients, respectively, preceding the onset of motor symptoms. In our study, we found that the odor identification test successfully differentiated healthy controls and iRBD patients, who are at higher risk for developing PD [9]. Additionally, according to the research criteria for prodromal PD made by International Parkinson and Movement Disorder Society (MDS) Task Force [41], both olfactory loss and PSG-proven RBD have a relatively high positive likely ratio (LR) in diagnosing prodromal PD, such that one person with positive scores in both of these areas will definitely meet the criteria for prodromal PD, if there are no other major markers to influence the diagnosis. This evidence indicates that the combination of the olfactory test and PSG-proven RBD may differentiate prodromal PD patients and healthy people. Considering that the olfactory test is cheap and easy to perform, although it has limitations for its low specificity, we might still screen people at high risk of developing PD with the olfactory test in combination with RBD, and then make a formal diagnosis with an examination whose specificity is high, such as dopamine transporter imaging [42] and detection of α-synuclein oligomers in cerebrospinal fluid [43], making early intervention possible.

Even though it has potential in the diagnosis of iRBD and PD, the Sniffin’ Sticks test still has unsatisfactory components to improve or adjust. In our study, there was no significant difference among the three study groups in identification rates of cinnamon or cloves, which could possibly be explained by the unfamiliarity of these odors to Chinese patients. In addition, there have been previous studies suggesting that a brief test based on the identification of three or five odors could achieve an acceptable specificity and sensitivity [44, 45]. Thus, we selected coffee, orange, mint, pineapple and fish to compose a brief odor identification test. Compared with the Sniffin’ Sticks test, the 5-item set has lower sensitivity but higher specificity for separating iRBD patients from controls, with the same sensitivity and higher specificity in separating PD and controls. Actually, the principle of the five odor selection is getting the accuracy of PD diagnosis as high as possible and making sure that all of these odors are familiar to the Chinese population; theoretically, the accuracy of differentiating PD patients from controls using the brief test will not be much lower than that observed with the longer Sniffin’ Sticks test. However, relative to the SS-12 test, there is a reduction in the sensitivity in discriminating iRBD patients from controls with the 5-item set. This difference confirms that the heterogeneity of RBD is greater [11]. Because only a proportion of RBD patients will develop PD [33], olfactory impairment in RBD patients is more variable than in PD. While the specificity of PD diagnosis increases with the brief test, more RBD patients will be outside the range setting for hyposmia in PD, which can also account for the improved sensitivity and decreased specificity in separating PD and iRBD patients with the brief test. We observed similar results in the validation test with the 5-item set in new groups composed of 35 healthy controls, 35 iRBD patients and 35 PD patients matched by age and sex. According to the validation results, the brief test can greatly improve the specificity in diagnosing PD, especially when separating PD and iRBD, and can be time-saving and suitable for a quick screen or for making a diagnosis when combined with other methods.

There are some limitations of our study. We accessed the olfactory function of iRBD patients to study prodromal PD patients, but some of the iRBD patients will not develop PD in the future. A substantial proportion of them may develop DLB (26.9%), and a small proportion of them may develop MSA (3.85%) or remain disease free [11]. Current studies suggested that, patients with DLB had marked olfaction dysfunction [46, 47] as PD patients, while patients with MSA had relatively intact olfaction [48, 49]. This study is obviously limited by being only cross-sectional in nature, in which the outcome of the follow up was not obtained to confirm the diagnostic conversion of RBD. Prospective studies are worthy of further investigation and are currently underway. Another limitation of our study is that we did not investigate patients less than 55 years of age, for they may have different pathogenesis. Thus, our results could not be generalized to these patients. Additionally, because of the lack of some information, the study groups in the validation were not matched by education.

## Conclusions

In conclusion, we found that olfactory impairment is common in iRBD patients, but to a lesser degree than that in PD. The difference in odor identification between iRBD patients and PD patients may be due to the heterogeneity of iRBD patients, with follow-up study being needed for further investigation. Based on the results, the Sniffin’ Sticks screening 12 test could be a useful tool for differentiating iRBD patients from healthy people and, in combination with other tests such as PSG, it could be useful for screening Chinese people at high-risk of PD. Furthermore, a brief odor identification test composed of coffee, orange, mint, pineapple and fish shows similar accuracy and is less time consuming than the Sniffin’ Sticks test, implying that it could be of value for application in China.

## Supporting Information

S1 Table Additional Clinical Information in the Odor Identification Test. (XLSX)

S2 Table Effect Sizes of the variants in the Odor Identification Test. (XLSX)

S1 Text Correlation Analysis between Olfactory Impairments and Potential Determinants. (DOCX)

## References

[1] Jellinger KA. Neuropathobiology of non-motor symptoms in Parkinson disease. J Neural Transm (Vienna). 2015;122(10):1429–40. 10.1007/s00702-015-1405-5 . 25976432

[2] Zhang ZX, Roman GC, Hong Z, Wu CB, Qu QM, Huang JB, et al Parkinson's disease in China: prevalence in Beijing, Xian, and Shanghai. Lancet. 2005;365(9459):595–7. 10.1016/S0140-6736(05)17909-4 . 15708103

[3] Tolosa E, Gaig C, Santamaria J, Compta Y. Diagnosis and the premotor phase of Parkinson disease. Neurology. 2009;72(7 Suppl):S12–20. 10.1212/WNL.0b013e318198db11 . 19221308

[4] Doty RL. Olfactory dysfunction in Parkinson disease. Nat Rev Neurol. 2012;8(6):329–39. 10.1038/nrneurol.2012.80 . 22584158

[5] Schenck CH, Bundlie SR, Patterson AL, Mahowald MW. Rapid eye movement sleep behavior disorder. A treatable parasomnia affecting older adults. JAMA. 1987;257(13):1786–9. . 3820495

[6] medicine Aaos. International Classification of Sleep Disorders. 2nd ed. Westchester, editor2005.

[7] Chaudhuri KR, Naidu Y. Early Parkinson's disease and non-motor issues. J Neurol. 2008;255 Suppl 5:33–8. 10.1007/s00415-008-5006-1 . 18787880

[8] Comella CL, Nardine TM, Diederich NJ, Stebbins GT. Sleep-related violence, injury, and REM sleep behavior disorder in Parkinson's disease. Neurology. 1998;51(2):526–9. Epub 1998/08/26. . 9710029 10.1212/wnl.51.2.526

[9] Postuma RB, Gagnon JF, Vendette M, Fantini ML, Massicotte-Marquez J, Montplaisir J. Quantifying the risk of neurodegenerative disease in idiopathic REM sleep behavior disorder. Neurology. 2009;72(15):1296–300. Epub 2008/12/26. 10.1212/01.wnl.0000340980.19702.6e ; PubMed Central PMCID: PMCPmc2828948. 19109537 PMC2828948

[10] Schenck CH, Boeve BF, Mahowald MW. Delayed emergence of a parkinsonian disorder or dementia in 81% of older men initially diagnosed with idiopathic rapid eye movement sleep behavior disorder: a 16-year update on a previously reported series. Sleep Med. 2013;14(8):744–8. 10.1016/j.sleep.2012.10.009 . 23347909

[11] Iranzo A, Fernandez-Arcos A, Tolosa E, Serradell M, Molinuevo JL, Valldeoriola F, et al Neurodegenerative Disorder Risk in Idiopathic REM Sleep Behavior Disorder: Study in 174 Patients. PLOS One. 2014;9(2):e89741 10.1371/journal.pone.0089741 24587002 PMC3935943

[12] Stiasny-Kolster K, Doerr Y, Moller JC, Hoffken H, Behr TM, Oertel WH, et al Combination of 'idiopathic' REM sleep behaviour disorder and olfactory dysfunction as possible indicator for alpha-synucleinopathy demonstrated by dopamine transporter FP-CIT-SPECT. Brain: a journal of neurology. 2005;128(Pt 1):126–37. 10.1093/brain/awh322 . 15548552

[13] Fantini ML, Postuma RB, Montplaisir J, Ferini-Strambi L. Olfactory deficit in idiopathic rapid eye movements sleep behavior disorder. Brain research bulletin. 2006;70(4–6):386–90. Epub 2006/10/10. 10.1016/j.brainresbull.2006.07.008 . 17027774

[14] Postuma RB, Lang AE, Massicotte-Marquez J, Montplaisir J. Potential early markers of Parkinson disease in idiopathic REM sleep behavior disorder. Neurology. 2006;66(6):845–51. 10.1212/01.wnl.0000203648.80727.5b . 16567700

[15] Miyamoto T, Miyamoto M, Iwanami M, Suzuki K, Inoue Y, Hirata K. Odor identification test as an indicator of idiopathic REM sleep behavior disorder. Movement disorders: official journal of the Movement Disorder Society. 2009;24(2):268–73. Epub 2008/10/31. 10.1002/mds.22361 . 18972547

[16] Postuma RB, Gagnon JF, Vendette M, Montplaisir JY. Markers of neurodegeneration in idiopathic rapid eye movement sleep behaviour disorder and Parkinson's disease. Brain: a journal of neurology. 2009;132(Pt 12):3298–307. 10.1093/brain/awp244 . 19843648

[17] Iwanami M, Miyamoto T, Miyamoto M, Hirata K, Takada E. Relevance of substantia nigra hyperechogenicity and reduced odor identification in idiopathic REM sleep behavior disorder. Sleep medicine. 2010;11(4):361–5. 10.1016/j.sleep.2009.12.006 . 20223708

[18] Miyamoto T, Miyamoto M, Iwanami M, Hirata K. Olfactory dysfunction in Japanese patients with idiopathic REM sleep behavior disorder: comparison of data using the university of Pennsylvania smell identification test and odor stick identification test for Japanese. Movement disorders: official journal of the Movement Disorder Society. 2010;25(10):1524–6. 10.1002/mds.23170 . 20568095

[19] Miyamoto T, Miyamoto M, Iwanami M, Hirata K, Kobayashi M, Nakamura M, et al Olfactory dysfunction in idiopathic REM sleep behavior disorder. Sleep Medicine. 2010;11(5):458–61. 10.1016/j.sleep.2009.09.013 . 20378403

[20] Shin HY, Joo EY, Kim ST, Dhong HJ, Cho JW. Comparison study of olfactory function and substantia nigra hyperechogenicity in idiopathic REM sleep behavior disorder, Parkinson's disease and normal control. Neurological sciences: official journal of the Italian Neurological Society and of the Italian Society of Clinical Neurophysiology. 2013;34(6):935–40. 10.1007/s10072-012-1164-0 . 22843227

[21] Chen W, Chen S, Kang WY, Li B, Xu ZM, Xiao Q, et al Application of odor identification test in Parkinson's disease in China: a matched case-control study. J Neurol Sci. 2012;316(1–2):47–50. 10.1016/j.jns.2012.01.033 . 22364958

[22] Hummel T. 'Sniffin' Sticks': Olfactory Performance Assessed by the Combined Testing of Odor Identification, Odor Discrimination and Olfactory Threshold. Chemical Senses. 1997;22(1):39–52. 9056084 10.1093/chemse/22.1.39

[23] Antsov E, Silveira-Moriyama L, Kilk S, Kadastik-Eerme L, Toomsoo T, Lees A, et al Adapting the Sniffin' Sticks olfactory test to diagnose Parkinson's disease in Estonia. Parkinsonism & related disorders. 2014;20(8):830–3. 10.1016/j.parkreldis.2014.04.012 . 24792992

[24] Iijima M, Kobayakawa T, Saito S, Osawa M, Tsutsumi Y, Hashimoto S, et al Smell Identification in Japanese Parkinson's Disease Patients: Using the Odor Stick identification Test for Japanese Subjects. Internal Medicine. 2008;47(21):1887–92. 10.2169/internalmedicine.47.1345 18981632

[25] Hummel T, Konnerth CG, Rosenheim K, Kobal G. Screening of olfactory function with a four-minute odor identification test: reliability, normative data, and investigations in patients with olfactory loss. Ann Otol Rhinol Laryngol. 2001;110(10):976–81. . 11642433 10.1177/000348940111001015

[26] Iranzo A, Santamaria J, Tolosa E. The clinical and pathophysiological relevance of REM sleep behavior disorder in neurodegenerative diseases. Sleep Med Rev. 2009;13(6):385–401. 10.1016/j.smrv.2008.11.003 . 19362028

[27] Hughes AJ, Daniel SE, Kilford L, Lees AJ. Accuracy of clinical diagnosis of idiopathic Parkinson's disease: a clinico-pathological study of 100 cases. J Neurol Neurosurg Psychiatry. 1992;55(3):181–4. 1564476 10.1136/jnnp.55.3.181 PMC1014720

[28] Postuma RB, Arnulf I, Hogl B, Iranzo A, Miyamoto T, Dauvilliers Y, et al A single-question screen for rapid eye movement sleep behavior disorder: a multicenter validation study. Movement disorders: official journal of the Movement Disorder Society. 2012;27(7):913–6. Epub 2012/06/26. 10.1002/mds.25037 ; PubMed Central PMCID: PMCPmc4043389. 22729987 PMC4043389

[29] Goetz CG, Poewe W, Rascol O, Sampaio C, Stebbins GT, Counsell C, et al Movement Disorder Society Task Force report on the Hoehn and Yahr staging scale: status and recommendations. Mov Disord. 2004;19(9):1020–8. 10.1002/mds.20213 . 15372591

[30] Zhang MY, Katzman R, Salmon D, Jin H, Cai GJ, Wang ZY, et al The prevalence of dementia and Alzheimer's disease in Shanghai, China: impact of age, gender, and education. Ann Neurol. 1990;27(4):428–37. 10.1002/ana.410270412 . 2353798

[31] Joo YH, Hwang SH, Han KD, Seo JH, Kang JM. Relationship between olfactory dysfunction and suicidal ideation: The Korea National Health and Nutrition Examination Survey. American journal of rhinology & allergy. 2015;29(4):268–72. Epub 2015/07/15. 10.2500/ajra.2015.29.4194 . 26163247

[32] Postuma RB, Gagnon JF, Vendette M, Desjardins C, Montplaisir JY. Olfaction and color vision identify impending neurodegeneration in rapid eye movement sleep behavior disorder. Annals of neurology. 2011;69(5):811–8. Epub 2011/01/20. 10.1002/ana.22282 . 21246603

[33] Gagnon J-F, Postuma RB, Mazza S, Doyon J, Montplaisir J. Rapid-eye-movement sleep behaviour disorder and neurodegenerative diseases. The Lancet Neurology. 2006;5(5):424–32. 10.1016/s1474-4422(06)70441-0 16632313

[34] Hubbard PS, Esiri MM, Reading M, McShane R, Nagy Z. Alpha-synuclein pathology in the olfactory pathways of dementia patients. Journal of anatomy. 2007;211(1):117–24. 10.1111/j.1469-7580.2007.00748.x 17553102 PMC2375794

[35] Kikuchi A, Baba T, Hasegawa T, Sugeno N, Konno M, Takeda A. Differentiating Parkinson's disease from multiple system atrophy by [123I] meta-iodobenzylguanidine myocardial scintigraphy and olfactory test. Parkinsonism & related disorders. 2011;17(9):698–700. 10.1016/j.parkreldis.2011.07.011 . 21840242

[36] Suzuki M, Hashimoto M, Yoshioka M, Murakami M, Kawasaki K, Urashima M. The odor stick identification test for Japanese differentiates Parkinson's disease from multiple system atrophy and progressive supra nuclear palsy. BMC Neurol. 2011;11:157 10.1186/1471-2377-11-157 22192419 PMC3297535

[37] Hawkes CH, Del Tredici K, Braak H. A timeline for Parkinson's disease. Parkinsonism Relat Disord. 2010;16(2):79–84. 10.1016/j.parkreldis.2009.08.007 . 19846332

[38] Wood H. Parkinson disease: 18F-DTBZ PET tracks dopaminergic degeneration in patients with Parkinson disease. Nat Rev Neurol. 2014;10(6):305 Epub 2014/05/21. 10.1038/nrneurol.2014.81 . 24840973

[39] De Cock VC, Vidailhet M, Leu S, Texeira A, Apartis E, Elbaz A, et al Restoration of normal motor control in Parkinson's disease during REM sleep. Brain: a journal of neurology. 2007;130(Pt 2):450–6. 10.1093/brain/awl363 . 17235126

[40] Diederich NJ, Vaillant M, Mancuso G, Lyen P, Tiete J. Progressive sleep 'destructuring' in Parkinson's disease. A polysomnographic study in 46 patients. Sleep medicine. 2005;6(4):313–8. 10.1016/j.sleep.2005.03.011 . 15946897

[41] Berg D, Postuma RB, Adler CH, Bloem BR, Chan P, Dubois B, et al MDS research criteria for prodromal Parkinson's disease. Movement disorders: official journal of the Movement Disorder Society. 2015;30(12):1600–11. 10.1002/mds.26431 . 26474317

[42] Jennings DL, Seibyl JP, Oakes D, Eberly S, Murphy J, Marek K. (123I) beta-CIT and single-photon emission computed tomographic imaging vs clinical evaluation in Parkinsonian syndrome: unmasking an early diagnosis. Arch Neurol. 2004;61(8):1224–9. 10.1001/archneur.61.8.1224 . 15313838

[43] Tokuda T, Qureshi MM, Ardah MT, Varghese S, Shehab SA, Kasai T, et al Detection of elevated levels of alpha-synuclein oligomers in CSF from patients with Parkinson disease. Neurology. 2010;75(20):1766–72. 10.1212/WNL.0b013e3181fd613b . 20962290

[44] Christian Mueller BR. A new procedure for the short screening of olfactory function using five items from the “Sniffin’ Sticks”identification test kit. American Journal of Rhinology. 2006;20(1):113–6. 16539306

[45] Hummel T, Pfetzing U, Lotsch J. A short olfactory test based on the identification of three odors. J Neurol. 2010;257(8):1316–21. 10.1007/s00415-010-5516-5 . 20232208

[46] Sato T, Hanyu H, Kume K, Takada Y, Onuma T, Iwamoto T. Difference in olfactory dysfunction with dementia with lewy bodies and Alzheimer's disease. J Am Geriatr Soc. 2011;59(5):947–8. Epub 2011/05/17. 10.1111/j.1532-5415.2011.03380.x . 21568971

[47] Williams SS, Williams J, Combrinck M, Christie S, Smith AD, McShane R. Olfactory impairment is more marked in patients with mild dementia with Lewy bodies than those with mild Alzheimer disease. J Neurol Neurosurg Psychiatry. 2009;80(6):667–70. Epub 2009/05/19. 10.1136/jnnp.2008.155895 . 19448090

[48] Glass PG, Lees AJ, Mathias C, Mason L, Best C, Williams DR, et al Olfaction in pathologically proven patients with multiple system atrophy. Mov Disord. 2012;27(2):327–8. Epub 2011/09/29. 10.1002/mds.23972 . 21953531

[49] Goldstein DS, Holmes C, Bentho O, Sato T, Moak J, Sharabi Y, et al Biomarkers to detect central dopamine deficiency and distinguish Parkinson disease from multiple system atrophy. Parkinsonism Relat Disord. 2008;14(8):600–7. Epub 2008/03/08. 10.1016/j.parkreldis.2008.01.010 18325818 PMC2650101

