# Supplementary material for: Odor Identification Test in Idiopathic REM-Behavior Disorder and Parkinson's Disease in China
Source: PLoS One. 2016 Aug 2;11(8):e0160199. doi: 10.1371/journal.pone.0160199 (PMC4970766; doi:10.1371/journal.pone.0160199)
Supplement: S1 Text — (DOCX) [file pone.0160199.s003.docx]

S1 Text. Correlation Analysis between Olfactory Impairments and Potential Determinants.

1. PD patients:

The data didn’t distribute normally. Thus spearman rank correlation was conducted.

|  | | | OL |
| --- | --- | --- | --- |
| Spearman's rho | OL | Correlation Coefficient | 1.000 |
|  |  | Sig. (2-tailed) | . |
|  |  | N | 54 |
|  | SEX | Correlation Coefficient | .121 |
|  |  | Sig. (2-tailed) | .385 |
|  |  | N | 54 |
|  | AGE | Correlation Coefficient | -.211 |
|  |  | Sig. (2-tailed) | .125 |
|  |  | N | 54 |
|  | HY | Correlation Coefficient | -.019 |
|  |  | Sig. (2-tailed) | .891 |
|  |  | N | 54 |
|  | UPDRS | Correlation Coefficient | -.266 |
|  |  | Sig. (2-tailed) | .054 |
|  |  | N | 53 |

Sex and olfactory impairments:

rho=-0.121, P=0.385>0.05 .There is no significant difference.

Age and olfactory impairments:

rho=-0.211, P=0.125>0.05 .There is no significant difference.

H&Y and olfactory impairments:

rho=-0.019, P=0.891>0.05. There is no significant difference.

UPDRS-III scores and olfactory impairments:

rho=-0.266, P=0.054>0.05. There is no significant difference.

2. iRBD patients:

The data didn’t distribute normally. Thus spearman rank correlation was conducted.

|  | | | OL |
| --- | --- | --- | --- |
| Spearman's rho | OL | Correlation Coefficient | 1.000 |
|  |  | Sig. (2-tailed) | . |
|  |  | N | 54 |
|  | SEX | Correlation Coefficient | -.005 |
|  |  | Sig. (2-tailed) | .974 |
|  |  | N | 54 |
|  | AGE | Correlation Coefficient | -.304^*^ |
|  |  | Sig. (2-tailed) | .026 |
|  |  | N | 54 |
|  | chin_tonic | Correlation Coefficient | .076 |
|  |  | Sig. (2-tailed) | .610 |
|  |  | N | 48 |
|  | chin_phasic | Correlation Coefficient | .060 |
|  |  | Sig. (2-tailed) | .684 |
|  |  | N | 48 |
|  | limb_tonic | Correlation Coefficient | -.358^*^ |
|  |  | Sig. (2-tailed) | .013 |
|  |  | N | 48 |
|  | limb_phasic | Correlation Coefficient | -.402^**^ |
|  |  | Sig. (2-tailed) | .005 |
|  |  | N | 48 |
|  | PLMI | Correlation Coefficient | -.178 |
|  |  | Sig. (2-tailed) | .207 |
|  |  | N | 52 |
|  | AHI | Correlation Coefficient | .122 |
|  |  | Sig. (2-tailed) | .390 |
|  |  | N | 52 |

Sex and olfactory impairments:

rho=-0.005, P=0.974>0.05 .There is no significant difference.

Age and olfactory impairments:

rho=-0.304, P=0.026<0.05. There is significant difference.

Tonic EMG activity index/chin and olfactory impairments:

rho=0.076, P=0.610>0.05. There is no significant difference.

Phasic EMG activity index/chin and olfactory impairments:

rho=0.060, P=0.684>0.05. There is no significant difference.

Tonic EMG activity index/limb and olfactory impairments:

rho=-0.358, P=0.013<0.05. There is significant difference.

Phasic EMG activity index/limb and olfactory impairments:

rho=-0.402, P=0.005<0.05. There is significant difference.

PLMI and olfactory impairments:

rho=-0.178, P=0.207>0.05. There is no significant difference.

AHI and olfactory impairments:

rho=0.122, P=0.390>0.05. There is no significant difference.
